# 3D printing of cell-delivery scaffolds for tissue regeneration

**DOI:** 10.1093/rb/rbad032

**Published:** 2023-03-27

**Authors:** Jianmin Xue, Chen Qin, Chengtie Wu

**Affiliations:** State Key Laboratory of High Performance Ceramics and Superfine Microstructure, Shanghai Institute of Ceramics, Chinese Academy of Sciences, Shanghai 200050, People’s Republic of China; State Key Laboratory of High Performance Ceramics and Superfine Microstructure, Shanghai Institute of Ceramics, Chinese Academy of Sciences, Shanghai 200050, People’s Republic of China; State Key Laboratory of High Performance Ceramics and Superfine Microstructure, Shanghai Institute of Ceramics, Chinese Academy of Sciences, Shanghai 200050, People’s Republic of China; Center of Materials Science and Optoelectronics Engineering, University of Chinese Academy of Sciences, Beijing 100049, People’s Republic of China

**Keywords:** cell-delivery, 3D printing, tissue regeneration

## Abstract

Tissue engineering strategy that combine biomaterials with living cells has shown special advantages in tissue regeneration and promoted the development of regenerative medicine. In particular, the rising of 3D printing technology further enriched the structural design and composition of tissue engineering scaffolds, which also provided convenience for cell loading and cell delivery of living cells. In this review, two types of cell-delivery scaffolds for tissue regeneration, including 3D printed scaffolds with subsequent cell-seeding and 3D cells bioprinted scaffolds, are mainly reviewed. We devote a major part to present and discuss the recent advances of two 3D printed cell-delivery scaffolds in regeneration of various tissues, involving bone, cartilage, skin tissues etc. Although two types of 3D printed cell-delivery scaffolds have some shortcomings, they do have generally facilitated the exploration of tissue engineering scaffolds in multiple tissue regeneration. It is expected that 3D printed cell-delivery scaffolds will be further explored in function mechanism of seeding cells *in vivo*, precise mimicking of complex tissues and even organ reconstruction under the cooperation of multiple fields in future.

## Graphical abstract

**Figure:**
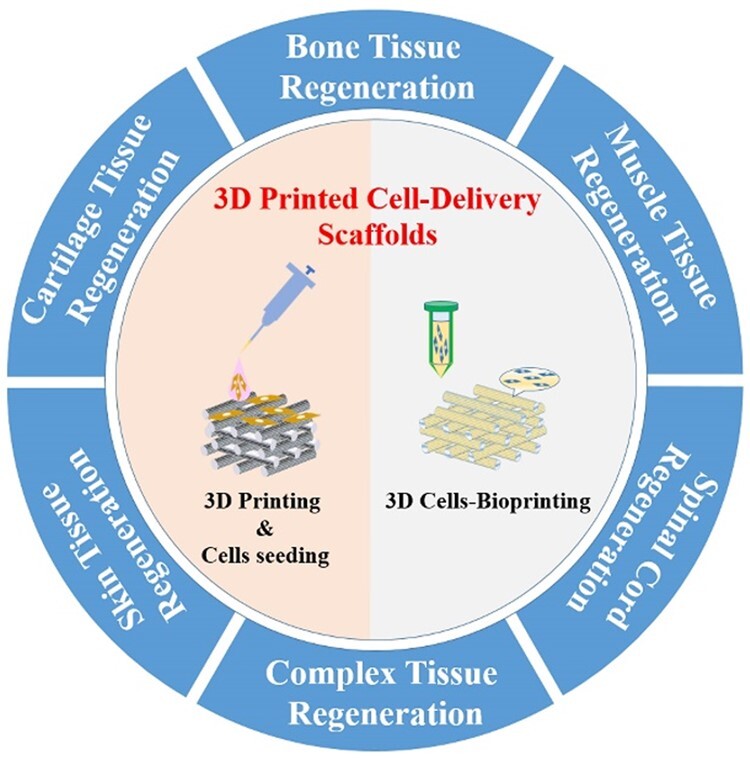

## Introduction

There are many people suffering from different degree of tissue damage caused by diseases, athletic injuries, natural disasters or other accidents, which greatly affects people’s physical health and quality of life. At present, although the autograft transplantation is still the ‘gold standard’ for tissue repairing, the secondary trauma and limited sources have become obstructions of its application. Besides, the allograft transplantation is also limited by the problems of immunological rejection and cross infection in clinic [1].

In the past decades, tissue engineering strategy of integrating biological scaffolds and living cells has been extensively studied and become a promising alternative for autograft and allograft transplantation [2]. Specifically, the tissue engineering scaffolds act as the artificial extracellular matrix (ECM) for supporting cell attachment, proliferation and delivery, finally creating functional constructs to replace/repair the injured tissues [3]. Therefore, tissue engineering scaffolds should possess several basic properties, such as excellent biocompatibility, sufficient mechanical support, good transportation capacity and suitable microenvironment for cells [4]. Seeding cell is another key factor of tissue engineering. As known, hypocellularity and injured cells with low metabolic activity are often occurred in defect area [5]. Therefore, supplementing living cells into the defects is a feasible way to guide new tissues formation [6, 7]. Notably, when deliver the cells, the cell type, cell distribution and cell retention should be fully considered [5]. In general, cells should be delivered with its composition and arrangement similar to natural tissues [8, 9]. Therefore, tissue engineering scaffolds should be well-designed to deliver the cells in a controlled manner.

In order to fabricate ideal cell-delivery scaffolds, various manufacture methods, such as lyophilization, electrospinning and 3D printing, have been applied to imitating the tissue structure [10–13]. Among these methods, 3D printing has attracted a lot of attention due to its advantages in imitating the 3D structure of target tissue. In particular, 3D bioprinting, a newly emerging method of 3D printing, shows unique capacity for achieving the precise spatial distribution of multiple cells and biomaterials [14–16]. The development of 3D printing technology definitely enhances the cell delivery and cell loading efficiency of scaffolds.

Typically, there are two approaches to achieve cell delivery by 3D printed scaffolds: (i) seeding cells after completing the fabrication of scaffolds; (ii) embedding cells in bioinks and molding in one-step through 3D bioprinting. The first method has friendly requirements for conditions and procedure of 3D printing process, but it is limited by low cell adhesion rate (or cell density) on the scaffolds. To overcome this problem, one of the effective strategies was employing hydrogels to encapsulate cells and then integrated them with 3D printed scaffolds [17, 18]. The second approach could significantly improve the cell loading efficiency and easily control the spatial distribution of multiple cells [19, 20]. However, 3D bioprinting method has certain requirements for the printing environment and parameters since cells are sensitive to inappropriate conditions, which means that the bioinks and the printing conditions should be detailedly designed in advance [21].

**Figure 1.**
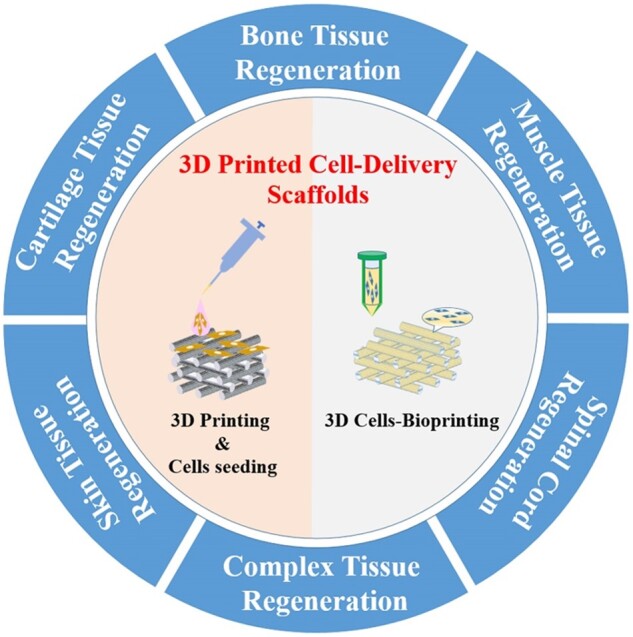
The classification and application of 3D printed cell-delivery scaffolds for tissue regeneration.

In this review, we summarized the recent progress of 3D printed cell-delivery scaffolds for tissue regeneration. We mainly focused on the two typical types of 3D printed cell-delivery scaffolds, 3D printed scaffolds with subsequent cell-seeding and 3D cells bioprinted ones, respectively (Fig. 1). Their application in different tissue regeneration, such as bone, cartilage, skin, muscle, nervous system and complex tissues, were discussed. Finally, we have highlighted challenges and proposed the future perspectives of 3D printed cell-delivery scaffolds for tissue regeneration. Notably, only the studies that have investigated the *in vivo* repair effects were included in this review.

## Traditional 3D printed scaffolds with cell seeding for tissue generation

In the past decades, 3D printing has become one of the most popular methods to fabricate tissue engineering scaffolds. As tissue engineering strategy has been more emphasized, the combination of 3D printed scaffold with cells has been deeply studied and facilitate functional tissue regeneration. In this section, we focused on the traditional cell-delivery approach of 3D printed scaffolds, *i.e.* seeding cells after completing the fabrication of scaffolds. Recent progresses of traditional 3D printed scaffolds with cell seeding in tissue generation will be discussed, mainly including bone tissue regeneration, cartilage tissue regeneration and complex tissue regeneration (Table 1).

**Table 1.** The advances of traditional 3D printed scaffolds with cell seeding for various tissues regeneration

Material	Cell type	Application	Reference
Copolymer poly (l-lactic acid-ε-caprolactone)/hydroxyapatite nanoparticles	Rat bone marrow mesenchymal stem cells	Vascularized bone regeneration	[22]
Hydroxyapatite/β-tricalcium phosphate/RGD peptide	Mesenchymal stem cells	Vascularized bone regeneration	[23]
Ca_2_MgSi_2_O_7_	Human bone marrow stem cells/human umbilical vein endothelial cells	Vascularized bone regeneration	[24]
β-tricalcium phosphate	Bone marrow-derived mesenchymal stem cells/Schwann cells	Innervated bone regeneration	[25]
Poly-(l-lactide-co-ε-caprolactone)/heparinized gelatin/transforming growth factor-β1	Human chondrocytes	Tracheal cartilage regeneration	[26]
Gelatin/hydroxyapatite	Human umbilical cord blood-derived mesenchymal stemcells	Articular cartilage regeneration	[27]
Poly(ε-caprolactone)	Adipose derived stem cells/chondrocytes	Cartilage regeneration	[17]
Polylactic acid	Human mesenchymal stem cells/Auricular chondrocytes	Auricular cartilage regeneration	[28]
Polycaprolactone/alginate	Mesenchymal stem cells/Chondrocytes	Osteochondral regeneration	[29]
Polylactic-co-glycolic acid	BMP-12-overexpressing mesenchymal stem cells	Tendon-bone regeneration	[30]
Polycaprolactone	Human gingival tissue multipotent mesenchymal stem/stromal cells	Skin tissue regeneration	[31]
Polycaprolactone	Neural crest stem cells derived Schwann cell progenitors	Peripheral nerve regeneration	[32]
Photoactive gelatin	Human-induced pluripotent stem cell-derived cardiomyocytes, smooth muscle cells, and endothelial cells	Cardiac muscle patch	[33]

### Bone tissue regeneration

Bone tissue, as the basic supporting and protective tissue of human body, is one of the most commonly transplanted parts in clinic [34, 35]. The enormous demands of bone transplantation also promote the rapid progress of bone substitutes both in the materials and manufacture techniques [36]. In the past decades, 3D printing has shown great potential in fabricating bone tissue engineering scaffolds due to their precise control of composition, structure or porosity of scaffolds [37]. In particular, inspired by the natural materials, such as lotus root, conch *etc.*, various 3D printed biomimetic scaffolds have been prepared in recent years, which greatly enriched the structural design and improved performance of bone tissue engineering materials [38, 39]. Herein, we mainly discussed 3D printed scaffolds with cell seeding for functionalized bone regeneration.

**Figure 2.**
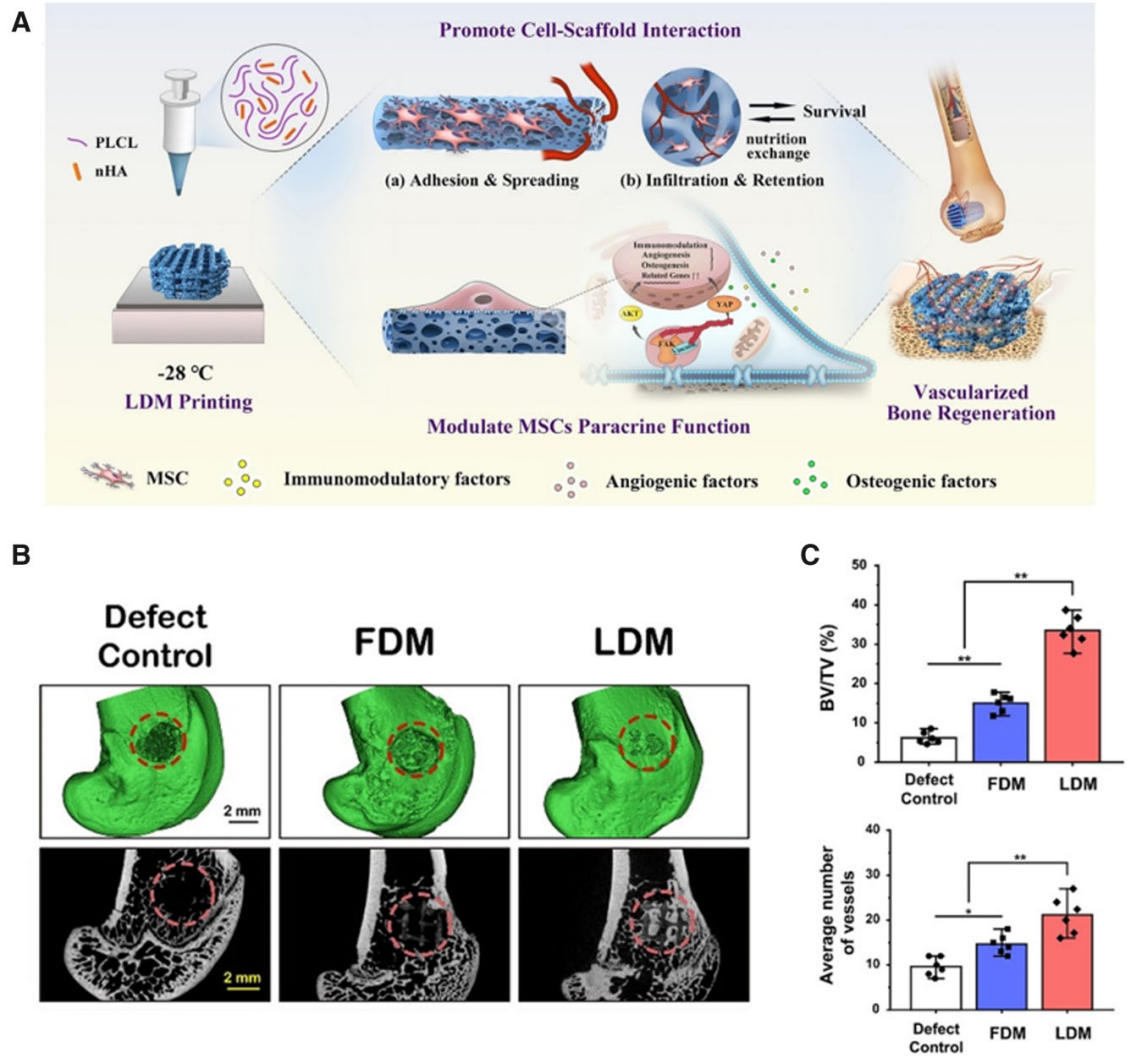
(**A**) Schematic of the design and application of sponge-like scaffolds fabricated by LDM 3D printing followed by freeze drying. (**B**) The micro-CT reconstruction images of LDM-printed cell-seeded scaffolds after implantation for 8 weeks. (**C**) Quantitative analysis of the newly formed bone (BV/TV) and average number of vessels of cell-seeded scaffolds after implantation for 8 weeks. (A–C) are adapted with permission from Ref. [22].

As well known, bone tissue is filled with blood vessels that are involved in physiological activities such as nutrient transport, metabolism and bone homeostasis maintenance [40, 41]. Consequently, it is important to promote the repair of bone tissue with blood vessels simultaneously for functionalized bone regeneration. Through the structure or composition regulation, 3D printed scaffolds could enhance the cell delivery capacity or activate the related cell behaviors, thus better achieving vascularized bone regeneration [42]. For example, 3D printing could be easily combined with other methods to construct macro/micro-structures for cell delivery or retention [43, 44]. The sponge-like scaffolds with hierarchical pores were fabricated through low-temperature deposition modeling (LDM) 3D printing followed by freeze drying [22]. Such hierarchical porous structures showed positive effect on the cell adhesion and retention and cell–material interactions. The rat bone marrow mesenchymal stem cells (rBMSCs)-seeded sponge-like scaffolds significantly enhanced the vascularized bone regeneration *in vivo* (Fig. 2). Besides, 3D printed scaffolds also could be modified to regulate the behavior of cells delivering by scaffolds. Wang *et al*. [23] used phage nanofibers binding with RGD peptide (RGD-phage) to modify 3D printed bioceramic scaffolds. The RGD-phage modified scaffolds were seeded with mesenchymal stem cells (MSCs) and then implanted into rat radius defects. The added RGD-phage is conducive to activate the migration, adhesion and differentiation of cells. Such MSCs-seeded scaffolds could induce the formation of blood vessels in newly formed bone.

The easy-constructed biomimetic structure for 3D printing scaffolds also paved the way for cell delivery of multiple cells. Zhang *et al*. [24] prepared biomimetic bioceramic scaffolds by mimicking the hierarchical structures, including Haversian canals and Volkmann canals of cortical bone structure and cancellous bone structure. Based on the bone-mimicking scaffold, they developed a multi-cellular delivery system by seeding MSCs on cancellous bone structure and loading endothelial cells in Haversian canals, which simulated the multiple bone resident cells in the microenvironment of bone tissue. The multiple cell-delivery scaffolds showed satisfied effect on the generation of bone and blood vessels *in vivo*, which provided a paradigm for 3D printed scaffolds for multicellular delivery and vascularized bone regeneration.

**Figure 3.**
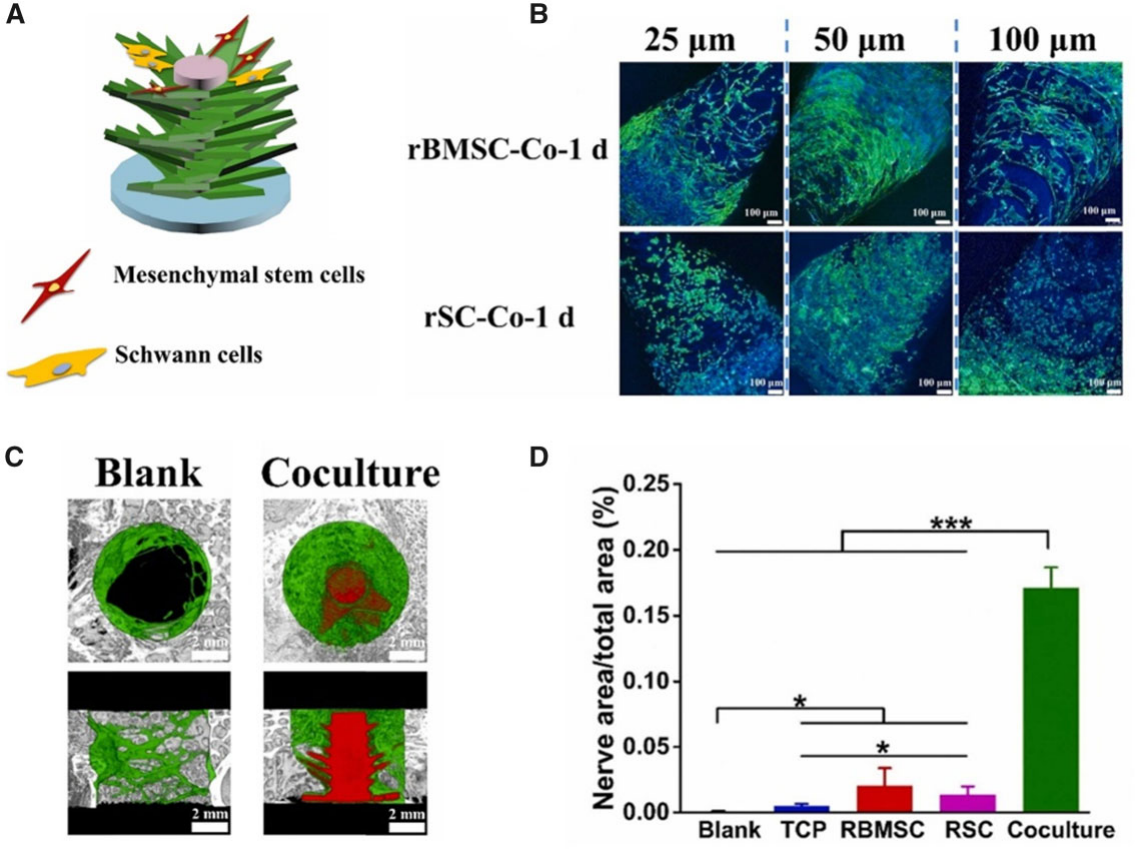
(**A**) Schematic of 3D printed tree-like scaffolds seeding with rBMSCs and rSCs. (**B**) The CLSM images of the cocultured rBMSCs and rSCs on scaffolds with different thickness of gradient surfaces for 1 day. (**C**) The micro-CT reconstruction images of rBMSCs/rSCs cocultured scaffolds after implantation for 8 weeks. (**D**) Quantitative analysis of nerve area/total area of rBMSCs/rSCs cocultured scaffolds after implantation for 8 weeks. (A–D) are adapted with permission from Ref. [25].

Sensory nerves and sympathetic nerves can participate in bone tissue regeneration through secreting neurotrophins and neuropeptides [45–47]. Therefore, 3D printed scaffolds were also expanded to innervated bone regeneration. Inspired by the phyllotaxis of plants, Zhang *et al*. [25] developed tree-like bioceramic scaffolds with multilayers of arranged leaves, providing ideal 3D platform for multicellular delivery and multiple cell-interactions (Fig. 3). The rabbit BMSCs and Schwann cells (SCs) were seeded on adjacent leaves to construct indirect contact co-cultured system. It was proved that the spatial architecture and gradient surface structure of tree-like scaffolds could influence the cell delivering and cell behaviors, while interactions between two types of cells significantly promoted *in vitro* osteogenic and neurogenic differentiation in co-cultured system. Moreover, such bioinspired scaffolds with co-cultured cells could simultaneously promote the growth of bone and nerves *in vivo*. This work not only achieved groundbreaking exploration of 3D printed cell-seeded bioceramic scaffolds in innervated bone regeneration, but also provided a paradigm for improving cell viability and the mutual talks of multiple cells by designing proper multiscale structures.

### Cartilage tissue regeneration

Cartilage tissue is a kind of tissue that cannot be ignored in the human body, which was widely found in joints, auricles and trachea [48]. Cartilage tissue has poor ability of nutrient nutrients transportation, since there is no blood vessel within it [49]. Therefore, once it gets injured, the damaged cartilage is hard to self-repair. To better treat the cartilage defects, new operation procedure, so called matrix-associated autologous chondrocyte implantation, has been proposed in clinic [50, 51]. In this method, the cultured cells are seeded on the matrix scaffold and then transplanted into the cartilage defect. It is actually a good practice to cartilage tissue engineering, which also encourages the study of 3D printed cell-seeded scaffolds in cartilage repair.

The most advantage of 3D printed scaffolds is that they can easily mimic natural cartilage tissue structures to prepare tissue-engineered constructs with irregular shape, which expanded the application field of cartilage tissue engineering from articular cartilage to auricular or trachea cartilage. For example, previous studies fabricated 3D printed scaffolds with ear shape for the delivering of chondrocytes, which achieved good effect on the reconstruction of auricle cartilage [17, 26, 27]. Furthermore, Park *et al*. [26] developed 3D printed hollow bellows scaffolds and assembled chondrocytes-containing functionalized sponges into grooves of scaffold for by mimicking the structure of trachea. Such tissue-engineered trachea possessed ideal ability to promote cartilage regeneration *in vivo* and showed similar mechanical performance with natural trachea, which provided novel tracheal substitute for the repair of circumferential tracheal defects.

Other efforts had also been made to explore different matrix components of 3D printed scaffolds with cell seeding for cartilage tissue engineering. Generally, the material source of cartilage tissue engineering scaffolds is mainly synthetic and natural polymers, such as collagen, gelatin, polylactic acid (PLA), poly(lactic-co-glycolic acid) (PLGA) or hyaluronic acid. Recently, Huang *et al*. [27] doped inorganic component of hydroxyapatite (HAP) in the gelatin to print scaffolds and then seeded MSCs on scaffolds for articular cartilage repair. The addition of HAP in scaffolds not only promoted the rheological properties, but also helped in the chondrogenic differentiation of MSCs and the *in vivo* cartilage repair.

For cartilage tissue engineering, chondrocytes or stem cells that can differentiate into chondrocytes are usually selected as the seed cells laden on the 3D printed scaffolds. Specially, the limited yields of isolated chondrocytes from cartilage tissue promoted the co-culture of chondrocytes with stem cells with chondrogenic potential. Jang *et al.* [17] demonstrated that the co-culture of adipose-derived stem cells (ASCs) with chondrocytes is beneficial to chondrogenic differentiation and cartilage regeneration through *in vitro* and *in vivo* experiments of ASCs/chondrocytes-laden 3D printed scaffolds. Dong *et al.* [28] used collagen to encapsulate human MSCs and auricular chondrocytes, and then seeded them on 3D printed PLA scaffolds for cartilage regeneration. After 6 months of implantation, the group with 10% cell ratio of chondrocytes can still effectively generate cartilage tissue, which was not different from high ratio of chondrocytes group (50%) in volume, histologic and compressive strength.

### Complex tissue regeneration

The complex tissues are composed of two or more tissue types with different properties and lineages. Some common examples of complex tissues include osteochondral tissue, tendon to bone tissue. Traditional cell-delivery 3D printed scaffolds not only could be used for the regeneration of single type of tissue, but also have practice on complex tissue regeneration. Bone-cartilage tissue is a typical complex tissue in human joints. Most of the articular cartilage damage will spread to the subchondral bone, forming the osteochondral defect. For osteochondral defect regeneration, it is necessary to repair cartilage, subchondral bone as well as the bone-cartilage interface simultaneously. To this end, Critchley *et al*. [29] designed bi-phasic 3D printed scaffolds for treatment of osteochondral defects. The bi-phasic scaffolds were loaded with MSCs-containing hydrogels below as ‘osseous phase’ while self-assembled MSC-chondrocyte layer on the top as ‘chondral phase’, providing an example for the design of 3D printed cell-seeded scaffolds for osteochondral regeneration. There are also some cases about 3D printed scaffolds for tendon-bone complex tissue regeneration. Chen *et al.* [30] fabricated bone morphogenic protein-overexpressing MSCs-seeded 3D printed PLGA scaffolds and selected rabbit rotator cuff model to evaluate the healing condition of tendon-bone tissue. *In vivo* results revealed that the cell-seeded scaffolds could promote fibrocartilage formation and collagen organization at tendon-bone interface, showing positive effect on tendon-bone tissue healing. In other respects, Lee *et al.* [52] were tried to investigate the application of 3D printed scaffolds seeding with cells on auricular cartilage-fat tissue regeneration. They printed ear shaped scaffolds and then seeded chondrocytes on auricle part and adipocytes on earlobe part, respectively. They verified that the chondrogenesis and adipogenesis were promoted simultaneously in co-cultured system, but further *in vivo* evaluation was not carried out in their work.

### Other tissues regeneration

Apart from the above tissues, traditional cell seeding 3D printed scaffolds also extend for other tissues regeneration, such as skin tissue, nervous system or muscle-related tissues. Achieving rapid skin wound healing with less scar tissue is still a hot topic in skin tissue regeneration. Combining 3D printed matrix material and cell delivery is a potential strategy to solve this problem. It is reported that 3D printed polycaprolactone scaffolds seeded with human gingival MSCs were developed and applied as biomimetic wound dressings [31]. Such cell-seeded scaffolds could promote the closure rate and reduce scar tissue area of full-thickness skin wounds after 6 weeks implantation. For nerve regeneration, it has certain requirements for mechanical support, porosity and aligned structures of nerve guidance conduits. He and Chen *et al*. [32] prepared 2D latticed scaffolds with desirable fibers orientation at long axis and circumferential axis through high-resolution electrohydrodynamic 3D printing method. The longitudinal fibers were designed to guide the alignment of cells while the circumferential fibers played effect on mechanical support. Then, the two-dimensional latticed scaffolds were seeded with neural crest stem cells derived SC progenitors and rolled up to multilayered cylinder scaffolds for peripheral nerve regeneration. The group of 3D printed scaffold with cells showed more myelinated axons, more orderly myelinated fibers and thicker myelin sheaths compared to the groups of cells or scaffolds alone.

Besides, traditional 3D printed scaffolds with cell seeding also showed potential to be used for cardiac muscle patch. Gao *et al*. [33] printed high-resolution scaffold by mimicking cardiac ECM structures and then seeded human-induced pluripotent stem cells on the scaffold to form the cardiac muscle patch. They successfully used novel multiphoton-excited 3D printing technique to prepare ECM-like scaffold with submicron resolution, which could not be achieved by conventional 3D printing technology and provided a potential method to fabricate fibrillar or mesh-like tissue structures. Moreover, it is proved that the cell-seeded cardiac muscle patch has positive effect on restoring cardiac function and eliminating the infarct size in murine model of myocardial infarction. Other efforts were also made to investigate the application of traditional cell seeding 3D printed scaffolds in skeletal muscle regeneration. However, these researches are limited to *in vitro* cell experiments while their biological properties *in vivo* still remain to be investigated [53, 54]. Considering 3D bioprinting technology has better progress in the field of skin, nerve and muscle regeneration, more related cases will be discussed in detail in the next section of 3D cells bioprinted scaffolds for tissue regeneration.

Altogether, traditional 3D printed scaffolds with cell seeding have been used for different tissue regeneration, exploring an effective way for multicellular delivery and functionalized tissue regeneration.

## 3D cells bioprinted scaffolds for tissue regeneration

As discussed above, the traditional 3D printed scaffolds seeding with cells can solve the problem of hypocellularity in the lesion area. However, the process of manually seeding cells makes it difficult to integrate multiple cells with specific spatial distribution, leading to the challenge of fully emulating the native tissues. As a coping strategy, 3D cell printing was developed and applied to construct the cellular system with biomimetic characteristics for improving the regenerative effects.

The 3D bioprinting technology has attracted more and more attention in tissue engineering field [55, 56]. Compared with the traditional cell-delivery approach (*i.e.* seeding cells after completing the fabrication of scaffolds), cell-delivery scaffolds prepared by 3D bioprinting could be more precisely to regulate the spatial distribution and density of multiple cells in one-step process [57, 58]. Moreover, because of its high degree of adjustability and accuracy, it is possible to produce more personalized cell-laden constructs via 3D bioprinting.

For designing the 3D cells bioprinted scaffolds, here are some of the key factors to consider: (i) The biomimetic structure. Understanding the macro and micro structure of the natural tissues and their ECM is critical to design the scaffolds with a biomimetic structure. Then, the structure can be designed via the 3D-modeling program, and the use of 3D printing technology can build those biomimetic structure accurately [19]. (ii) The appropriate cell types. The cell type should be relevant to the intended application. The cells that involved in the scaffolds should be beneficial for the repair of specific tissues, or be the important component of the natural tissues. Besides, the cell source should be considered, as some cells may be easier to obtain and expand in culture [6]. (iii) Material selection for bioinks. To design the materials used for bioinks, the balance of multiple properties, such as viscosity, cell viability and mechanical strength, should be well considered. The bioink materials must possess excellent biocompatibility to support the cell viability and proliferation. Besides, the bioinks should have suitable printability and mechanical strength to well maintain the 3D structure of the scaffolds. Moreover, bioink materials are best to possess bioactivities to induce the cells to achieve their physiological functions [59]. Therefore, the advances of 3D cells bioprinted scaffolds for various tissues regeneration were summarized as following (Table 2).

**Table 2.** The advances of 3D cells bioprinted scaffolds for various tissues regeneration

Material	Cell type	Application	Reference
Nanosilicates/gelatin/alginate hydrogel	Rat bone marrow mesenchymal stem cells	Bone regeneration	[60]
Nifedipine-loaded ethosome/laponite incorporated sodium alginate/methacrylated gelatin composite hydrogel	Bone mesenchymal stem cells	Bone regeneration	[61]
Deferoxamine-loaded ethosomes incorporated gelatin methacrylate/gellan gum methacrylate hybrid hydrogel	Bone mesenchymal stem cells	Bone regeneration	[62]
Gelatin methacrylate	Bone mesenchymal stem cells	Bone regeneration	[63]
Fibrin/γ-irradiated RGD-modified alginate/gelatin methacrylate	Human umbilical vein endothelial cells/human bone marrow stem/stromal cells	Vascularized bone regeneration	[64]
Gelatin methacrylate/Polylactic acid/polyethylene glycol	Bone mesenchymal stem cells/rat aortic endothelial cell lines	Vascularized bone regeneration	[65]
Calcium silicate nanowires/gelatin methacrylate	Rat bone mesenchymal stem cells/Schwann cells	Innervated bone regeneration	[66]
Gelatin/fibrinogen/hyaluronic acid/glycerol composite hydrogel	Bone mesenchymal stem cells	Articular cartilage	[67]
Gelatin methacrylate/N-(2-aminoethyl)-4-(4-(hydroxymethyl)-2-methoxy-5-nitrosophenoxy) butanamide linked hyaluronic acid	Human skin fibroblasts/human umbilical vein endothelial cells	Skin regeneration	[68]
Strontium silicate incorporated gellangum/alginate/methylcellulose composite hydrogel	Human dermal fibroblasts/human umbilical vein endothelial cells	Vascularized skin regeneration	[69]
Platelet-rich plasma incorporated alginate-gelatin composite hydrogel	Dermal fibroblasts/epidermal stem cells	Skin regeneration	[70]
Acellular dermal matrix/gelatin methacrylamide	Human umbilical vein endothelial cells/dermal fibroblasts/human skin keratinocyte line	Skin regeneration	[71]
Polyurethane/gelatin	Fibroblasts, keratinocytes/endothelial progenitor cells	Skin regeneration	[72]
Sodium alginate/photocurable PEG-fibrinogen	Muscle precursor cells	Muscle regeneration	[73]
Gelatin methacrylamide	Myoblasts/human adipose stem cells	Muscle regeneration	[74]
Polyethylene glycol/gelatin methacrylamide	Neural progenitor cells	Spinal cord regeneration	[75]
Chitosan/hyaluronic acid/matrigel	Neural stem cells	Spinal cord regeneration	[76]
β-Cyclodextrin/gelatin methacrylamide/small molecule OSMI-4	Neural stem cells	Spinal cord regeneration	[77]
Gelatin methacrylamide	Bone mesenchymal stem cells/Schwann cells	Spinal cord regeneration	[78]
Sodium alginate/gelatin/hydroxyapatite and sodium alginate/gelatin	Osteoblasts/chondrocytes	Osteochondral regeneration	[79]
Li–Mg–Si bioceramics gellan gum/alginate/methylcellulose composite hydrogel	Chondrocytes/placenta-derived mesenchymal stem cells	Osteochondral regeneration	[80]
Methacrylated hyaluronic acid/Polycaprolactone incorporating kartogenin/β-TCP	Bone marrow-derived mesenchymal stem cell	Osteochondral regeneration	[81]
Polycaprolactone/poly lactic-co-glycolic acid/hyaluronic acid	Pre-differentiated autologous adipose-derived mesenchymal stem cells	Tendon-bone regeneration	[82]
Decellularized cardiac extracellular matrix/gelatin methacrylamide	Human cardiac progenitor cells	Cardiac repair	[83]
Decellularized extracellular matrix	Human cardiac progenitor cells/human turbinate tissue-derived mesenchymal stem cells	Cardiac repair	[84]

### Bone and cartilage tissue regeneration

In recent years, 3D bioprinting technology has been widely used in the field of bone tissue engineering [85–87]. In general, BMSCs-containing bioinks were usually selected to construct 3D bioprinted scaffolds for bone tissue engineering. In addition, by changing the components of bioinks, such as adding bioactive components or loading drugs, 3D bioprinted scaffolds could regulate multiple cell behaviors, thus better promoting bone tissue regeneration [59]. For example, Liu *et al*. [60] constructed rBMSCs-containing bone tissue engineering scaffolds through 3D bioprinting. The nanosilicates (nSi) in the bioinks improved the printability and mechanical strength of bioinks, while promoted the osteogenic differentiation of rBMSCs. *In vivo* results showed that the bioprinted scaffolds could repair bone defects rapidly and safely in calvarial bone defect models of rats. Li *et al.* [61] developed BMSCs-laden scaffolds with capability of releasing nifedipine. The 3D bioprinted scaffolds could promote osteogenic differentiation of BMSCs by regulating sympathetic nervous with released nifedipine. When the scaffolds were implanted into the calvarial defects, they could effectively improve bone regeneration *in vivo*. Li *et al.* [62] printed a BMSCs-scaffold which could sustainedly release deferoxamine (DFO)-loaded ethosomes (Eth) for bone regeneration. The released DFO from the scaffolds could accelerate vascularization and osteogenesis. What’s more, the authors found that the 3D bioprinted scaffolds could accelerate vascularized bone regeneration after implanted into the calvarial defects by activating the hypoxia-inducible factor 1-α (HIF1-α) signaling pathway. Moreover, Xie *et al.* [63] designed a composite bioink based on BMSCs-encapsulated microgels that was able to *in situ* bioprinting. The scaffolds were *in situ* printed into the cranial defects and promoted bone regeneration, verifying their potential in clinical applications.

**Figure 4.**
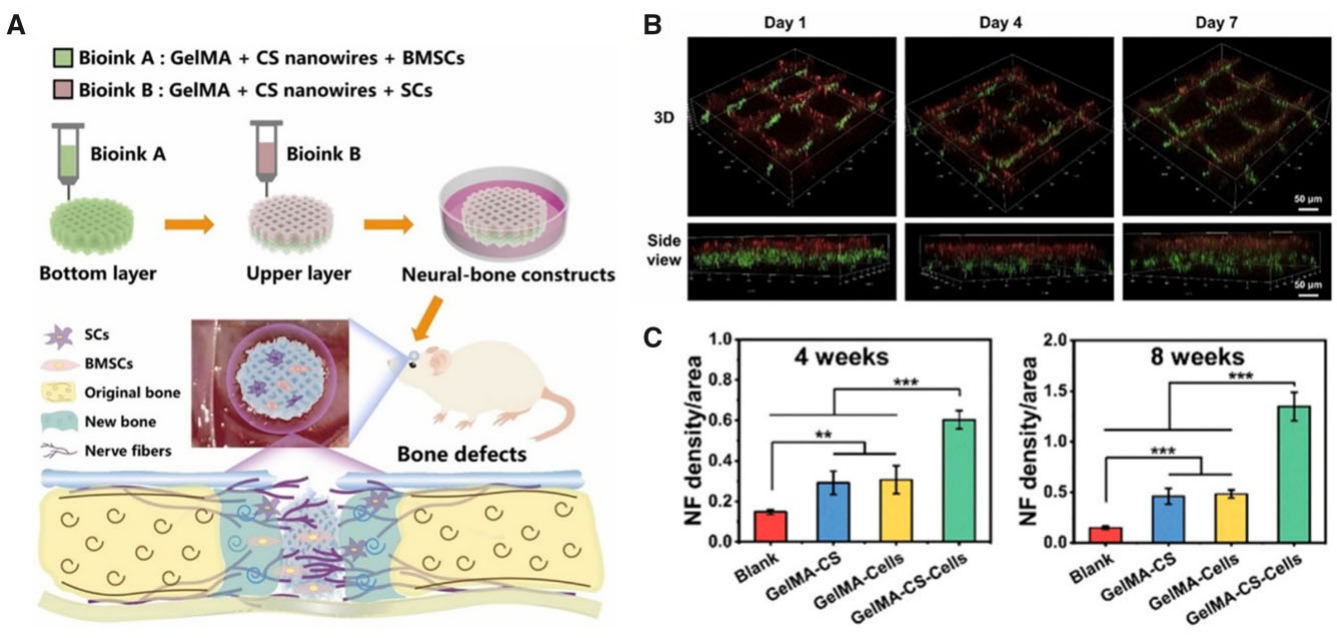
(**A**) Schematic illustration of the 3D bioprinted neural-bone constructs loaded BMSCs and SCs for innervated bone regeneration. The addition of CS nanowires and encapsulated cells synergistically promoted innervated bone regeneration. (**B**) The spatial distribution of BMSCs (bottom layer, green) and SCs (upper layer, red) within the 3D bioprinted neural-bone constructs. (**C**) Quantitative analysis of nerve fibers density after implantation for 4 and 8 weeks. (A–C) are adapted with permission from Ref. [66].

In addition to the above-mentioned scaffolds that only printed with MSCs, some researchers have developed scaffolds delivering multiple cells simultaneously for bone regeneration. For some large-sized bone defects, fully vascularization is critical in the regeneration process [88]. Therefore, several 3D bioprinted scaffolds with good vascularized properties were developed by simultaneously printing MSCs and endothelial cells. Nulty *et al.* [64] developed a bioink containing both BMSCs and human umbilical vein endothelial cell (HUVECs). The bioink was used to be printed with PCL scaffolds and implanted into femoral bone defects. The authors found that the scaffolds promoted the vascularization of the new tissues along with bone regeneration. Besides, Shen *et al.* [65] also developed scaffolds printed with both BMSCs and HUVECs. By applying an interlaced printing strategy, the HUVECs could distributed uniformly into the BMSCs-laden scaffolds. The authors claimed that this *in situ* EC bioprinting method led to greater cell seeding uniformity than the conventional cell-seeding method. In the calvarial defect models, the scaffolds promoted both bone regeneration and vascularization. Furthermore, the nerve networks play a significant guiding role in bone formation [89]. Zhang *et al.* [66] developed a biomimetic neural-bone construct delivering with BMSCs and SCs. With the inorganic calcium silicate nanowires incorporated, the cells bioprinted scaffolds could simultaneously support the osteogenic and neurogenic differentiation of encapsulated cells. Furthermore, by implanting the multicellular scaffolds into the cranial defects, the authors confirmed their abilities of achieving innervated bone regeneration (Fig. 4).

Apart from bone tissue regeneration, 3D cells bioprinted scaffolds have branched out tentatively into cartilage tissue regeneration. Sun *et al.* [67] printed growth differentiation factor 5 (GDF5)-conjugated BMSC-laden scaffold for repairing articular cartilage injury. With the incorporation of GDF5, the printed BMSCs showed stimulated differentiation toward chondrogenesis. The authors implanted the GDF5-conjugated BMSC-laden scaffolds into rabbit knee cartilage defects and demonstrated their better effects on cartilage regeneration.

### Skin tissue regeneration

As well known, skin tissue has layered structure consisting of epidermis, dermis and hypodermis. Taking advantage of the precise arrangement process of 3D bioprinting, biomimetic cell-containing constructs that simulated the layered structure of skin can be well prepared. In addition, due to the direct involvement of cells in the bioprinting process, the bioink materials used for printing are usually hydrogel biomaterials. Due to the similar mechanical properties to skin and excellent moisture retention property, hydrogel scaffolds with cells loaded are ideal candidates for skin regeneration [90]. Therefore, in recent years, many studies have developed biomimetic scaffolds printed with multiple cells to repair skin wounds.

Zhou *et al.* [68] developed biomimetic skin constructs by precisely printing human skin fibroblasts (HSFs) and HUVECs. The two types of cells could maintain layered distribution during the co-culture. The fabricated skin constructs containing two cells promoted the regeneration of full-thickness skin defects in a pig model. Ma *et al.* [69] developed biomimetic skin substitutes by fabricating a strontium silicate (SS)-containing multicellular system. The HUVECs and human dermal fibroblasts (HDFs) were printed as bi-layered distribution to mimic the structure of skin. With the stimulation of SS biomaterials, the angiogenic properties, which required for reconstruction of vascularized skin, were markedly promoted. Furthermore, the authors fully demonstrated the healing effects of the multicellular scaffolds for treating both acute and chronic wounds *via* three animal models. Zhao *et al.* [70] prepared platelet-rich plasma (PRPs)-containing 3D bioprinted constructs loaded dermal fibroblasts (DFs) and epidermal stem cells (ESCs). With the PRPs incorporation at concentration of 5%, the multicellular constructs were *in situ* printed on the wound beds and performed the most adequate reepithelization and fastest wound closure during a 21-day healing process.

**Figure 5.**
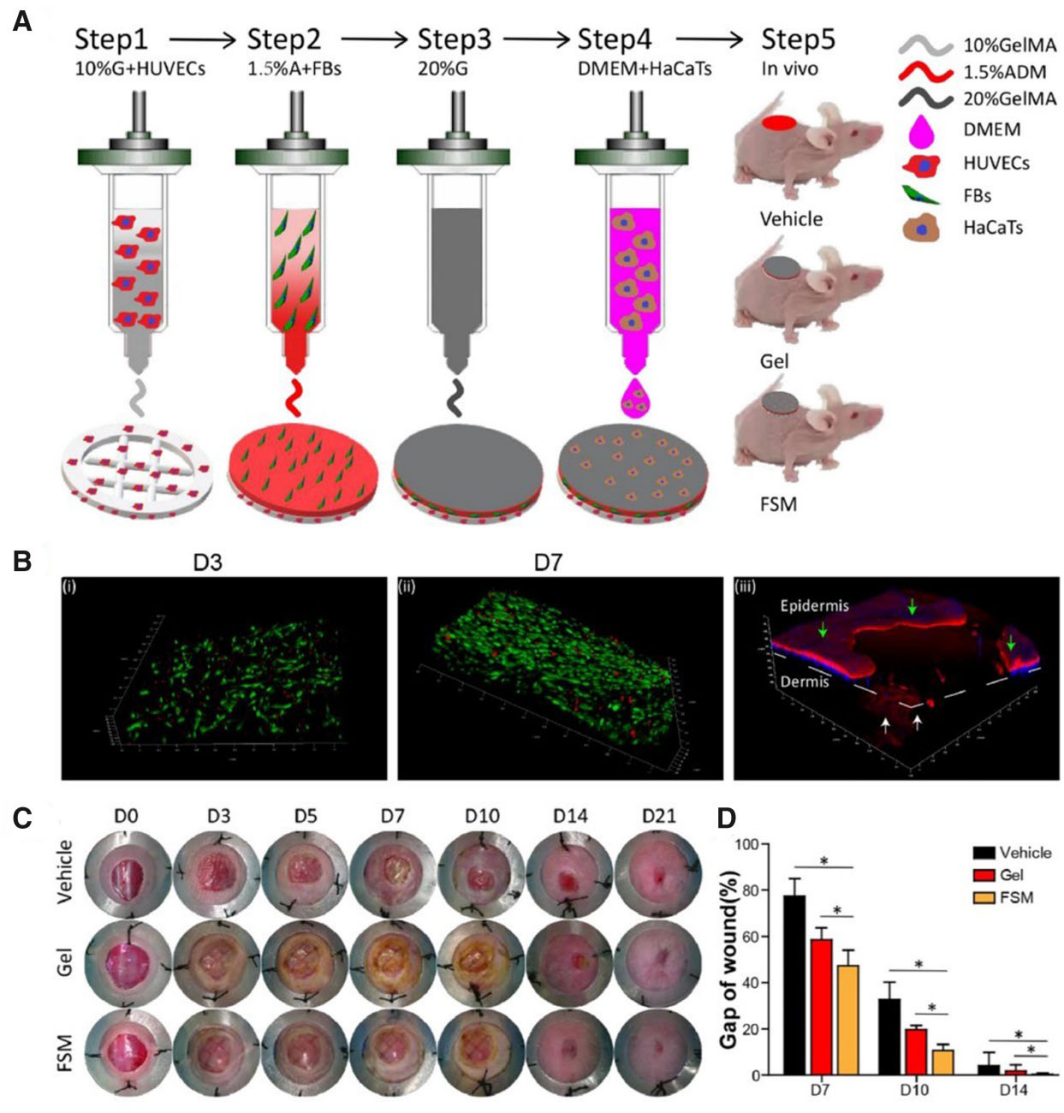
(**A**) Schematic illustration of the 3D bioprinted FSM containing multiple cells. (**B**) Live/dead assay of 3D bioprinted FSM after 3 days (i), 7 days (ii) of culture, (iii) multilayer structure of the FSM indicated by phalloidin/DAPI staining after 7 days of culture (epidermis was pointed by green arrow, dermis was pointed by white arrow). (**C**) The healing process of the wounds within 21 days. (**D**) Quantitative analysis of wound gap (*n* = 4–6). (A–D) are adapted with permission from Ref. [71].

In addition to the biomimetic skin constructs that printed with two types of cells, some researchers have developed constructs containing three types of cells to better simulate and repair the skin tissues. Jin *et al.* [71] proposed a functional skin model (FSM) with multilayer structure to simulate natural full-thickness skin (Fig. 5). Firstly, bioinks loaded HUVECs were printed as the vascular network for the bottom layer. Then, the fibroblasts were printed as the dermis layer. After that, human skin keratinocyte line was printed at the top of the scaffolds as an epidermal layer. After implanted into the wound beds, the 3D multicellular constructs could facilitate re-epithelization, dermal ECM secretion, vascularization and accelerate wound healing process. Wu *et al.* [72] developed curvilinear-bioprintable scaffolds loading three types of cells (fibroblasts, keratinocytes, and endothelial progenitor cells (EPCs)) to treat chronic wounds with irregular shape. After treating the large and irregular rat skin wounds for 28 days, the bioprinted tri-cell-laden scaffolds obviously promoted reepithelization and dermal repair as well as neovascularization and collagen production of the defects.

### Muscle tissue regeneration

Muscle tissues are constituted of muscle fibers which present regular spatial alignment. In order to manage the 3D arrangement of muscle cells and simulate the structure and microenvironment of natural skeletal muscles, 3D bioprinting has been applied to skeletal muscle tissue engineering. Costantini *et al.* [73] fabricated an artificial skeletal muscle tissue by 3D bioprinted muscle precursor cells (C2C12)-laden hydrogel with fiber structures. The bioprinted C2C12 could align toward the fibers direction and form multinucleated myotubes *in vitro*. After subcutaneously implanted into the nude mice, the 3D bioprinted artificial skeletal muscle formed striated myofibers, which indicated high degree of myotube maturation *in vivo*. Hwangbo *et al.* [74] developed cell-aligned constructs by bioprinting the C2C12 cells and human ASCs (hASCs) with an novel crosslinking system. They implanted the hASCs-laden constructs into the volumetric muscle defects of mouse and found that the cell-delivery constructs prepared by the modified printing process could accelerate the muscle regeneration.

### Spinal cord regeneration

Spinal cord injury (SCI) is a serious disease of central nervous system. The regeneration of spinal cord and the complete recovery of its function have not been achieved in clinic [91]. In recent years, biomimetic scaffolds with neural cells participated were found to be potential for repairing the damaged spinal cord. Therefore, several studies have developed 3D bioprinted scaffolds for spinal cord regeneration. Jacob *et al.* [75] bioprinted a neural progenitor cells (NPCs)-loaded scaffold by using microscale continuous projection printing method (μCPP) to repair the SCI. After implanted the scaffolds *in vivo*, the bioprinted NPCs extended axons and were well-integrated with the host axons. Thus, the bioprinted scaffolds were confirmed to support axon regeneration and improve the neural functions. Liu *et al.* [76] fabricated 3D bioprinted scaffolds loaded with neural stem cells (NSCs) based on novel bioinks consisting of chitosan, hyaluronic acid derivatives and matrigel. Then, the NSCs-laden scaffolds were implanted into the SCI defects of rats and were found to accelerate the axon regeneration and decrease the glial scar deposition. What’s more, the motor function of these rats was significantly promoted by the scaffolds. Recently, they also bioprinted an NSC-laden scaffold loading with OSMI-4, a small molecule O-GlcNAc transferase inhibitor [77]. The loaded small molecule could efficiently promote the neuron differentiation of NSCs, and thus achieve the high efficiency of SCI repair. In addition to the bioprinted scaffolds loaded with only one type of cells, there were several multicellular scaffolds developed for spinal cord regeneration. Wang *et al.* [78] developed 3D bioprinted scaffolds with specific spatial arrangements of BMSCs and SCs. It is demonstrated that such bi-cellular scaffolds facilitated motor function recovery of rats with their destroyed axons regenerated. Besides, Liu *et al.* [77] developed scaffolds loading NSCs in combination with oligodendrocytes for spinal cord regeneration by 3D bioprinting method. After the scaffolds were implanted into the completely transected spinal cord of rats, effective regeneration of nerve and motor function could be observed. To solve numerous problems occurred after SCI, such as neuronal atrophy and death, glial scar hyperplasia etc., the 3D bioprinted cell-delivery scaffolds which can supplement cells with specific spatial arrangements are undoubtedly the potential solution for treating SCI defect.

### Complex tissues and organs regeneration

The treatment of complex tissue defects can be challenging as it requires the integrated regeneration of multiple tissue types, each with its unique properties and requirements. Therefore, developing the cell-delivery scaffolds for treating complex tissues often involves the participation of multiple cells and biomaterials which are required to be orderly arranged in one single system. Benefitting from its advantages in designing the spatial arrangement of cells as well as bioink materials, 3D bioprinting is very applicable to construct the scaffolds for repairing the tissues that have gradient composition, such as bone-cartilage tissue and tendon-bone tissue.

**Figure 6.**
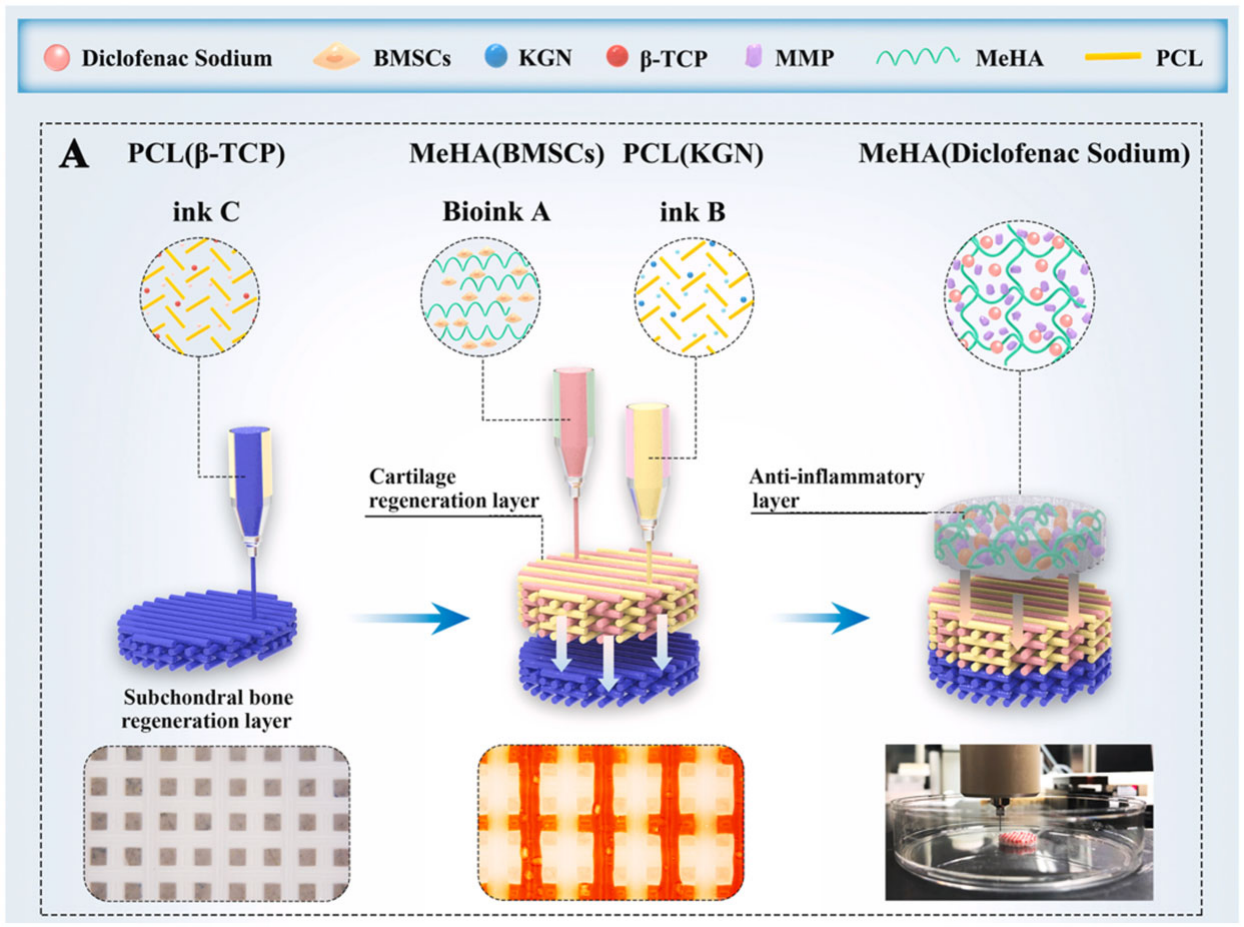
(**A**) Schematic illustration of the 3D bioprinted biomimetic scaffolds consisting of three different layers. The BMSCs were printed within the Middle layer to accelerate the cartilage regeneration. Adapted with permission from Ref. [81].

Several studies have developed the scaffolds printed with two kinds of cells to mimic the bilayered osteochondral tissues. By designing the cells and materials, respectively, fitted with cartilage and bone, Yang *et al.* [79] developed a biphasic scaffold for integrated regeneration of osteochondral bone. With the implantation of this scaffold loading osteoblasts and chondrocytes, improved repair of rabbit knee was achieved compared with the traditional tissue engineering methods. Qin *et al*. [80] developed an osteochondral-mimicking system with bi-layered structure containing chondrocytes and MSCs. By designing the bioinks incorporating silicate bioceramics and the spatial distribution of these two cells, the multicellular system simulated the anisotropic physiological characteristics of the osteochondral tissues, and could efficiently promote the integrated regeneration of osteochondral tissues. In addition to the multicellular scaffolds, researchers have developed kinds of scaffolds printed with single-cells to repair osteochondral defects. Aiming at treating the osteochondral defects induced by osteoarthritis (OA), Zhang *et al*. [81] developed biomimetic scaffolds consisting of three different layers, among which BMSCs were incorporated into the middle layer to promote the regeneration of cartilage tissues (Fig. 6). On the one hand, the scaffolds with biomimetic structure and composition could accelerate both the regeneration of cartilage and subchondral bone. On the other hand, by designing the matrix metalloproteinase-sensitive hydrogel with diclofenac sodium (DC)-delivery to be integrated on the scaffolds, the anti-inflammatory function of the scaffolds could be achieved. As a result, the composited scaffolds could efficiently repair the osteochondral defects in progressive OA joints of rats.

The tendon-to-bone interface is another typical complex tissue with gradient structure and composition. Researchers have developed 3D bioprinted biomimetic scaffolds to recapitulate the tendon-to-bone interface. Jiang *et al.* [82] bioprinted multilayered scaffolds containing the pre-differentiated autologous adipose-derived MSCs (ADMSCs) with different lineages. Firstly, the ADMSCs were pre-differentiated into three lineages, including tenogenic lineage, chondrogenic lineage, and osteogenic lineage. Then, they were printed as the three layers to construct the multilayered scaffolds with cell gradient. Compared with the acellular scaffolds, the ADMSCs-laden scaffolds with multilayered structure could significantly promote the tendon-to-bone regeneration by facilitating the cell communication and infiltration.

**Figure 7.**
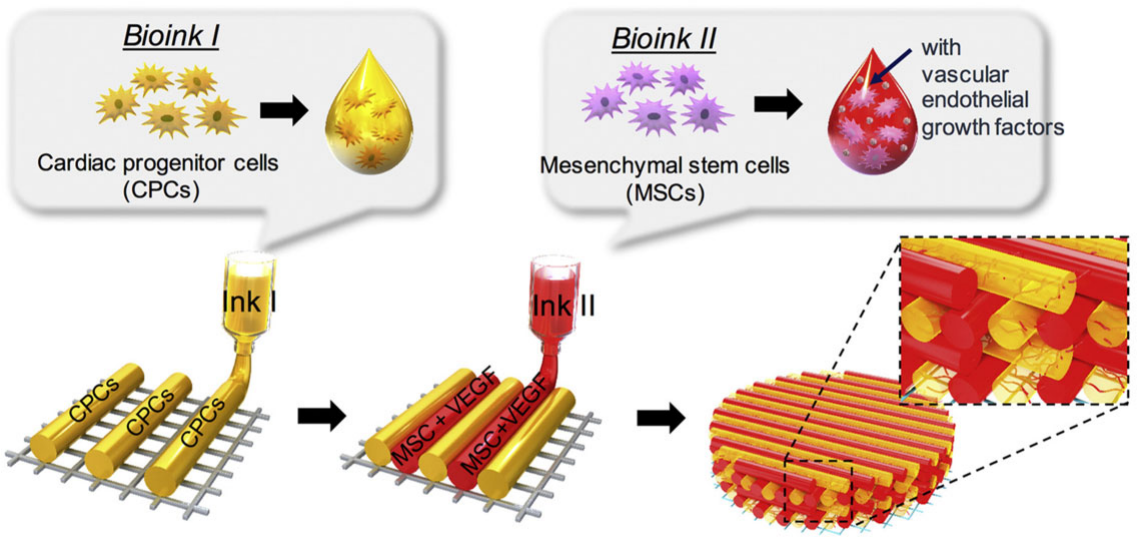
Printing process of the 3D pre-vascularized stem cell patch with two bioinks loaded with CPCs and MSCs. Adapted with permission from Ref. [84].

Beyond the application of repairing tissues with complex structure, 3D bioprinted cell-delivery scaffolds were also developed to repair organs. For the past few years, researchers set their sights on constructing cellular cardiac patch via 3D bioprinting method. Donald *et al.* [83] developed a cardiac patch consisting of GelMA hydrogel, decellularized cardiac ECM (cECM) and human cardiac progenitor cells (hCPCs). Taking advantages of the precise printing process, the cECM and hCPCs could be uniformly distributed in the cardiac patch. The composite cardiac patches were applied to treat the rat hearts suffering from myocardial ischemia and were found to promote vascularization and recovery of myocardial function. In addition, vascularization is crucial for cardiac repair. Therefore, Jang *et al.* [84] printed hCPCs together with MSCs to construct a 3D pre-vascularized stem cell patch. By alternatively printing two bioinks loaded with different cells, the multicellular patch with cells distributing in specific pattern could well prepared (Fig. 7). After implanted into the epicardium, this multicellular pre-vascularized patch promoted the vascularization and cardiac recovery simultaneously.

Accordingly, benefiting from its flexibility, 3D bioprinting method exhibited huge potential on constructing cell-delivery scaffolds for complex tissues and organs regeneration.

## Conclusion and prospect

In this review, we summarized the two types of 3D printed cell-delivery scaffolds and their application for tissue regeneration. The two preparation methods of 3D printed cell-delivery scaffolds have their own advantages and disadvantages. The way of seeding cells on 3D printed scaffolds allows wider range of biomaterials to be printed. What’s more, the cells will not be negatively affected by the printing process. Even so, it exhibited the obvious limitations of being unable to precisely distribute multiple cells. Although it is possible to seed cells in their place by designing specific structures, the operation is also complex.

The 3D cells bioprinting technology is evidently showing a better trend in developing cell-delivery scaffolds. It could be contributed to its integrated preparation process, which allows to control the cell distribution more precisely. However, the direct participation of living cells restricted the application of more biomaterials and the design of printing process. Therefore, it is necessary to optimize the printing conditions and cross-linking methods of bioink materials in order to balance the mechanical properties, printing properties and cell viability. To address these issues, more cooperation between materials scientists and mechanical engineers is required. It can be predicted that more functional tissue regenerative scaffolds were promising to be developed for reconstruction of multiple tissues and organs.

Based on the studies summarized in this review, it can be concluded that the cell-delivery scaffolds have great potential for the repair and regeneration of various tissue defects. In general, the delivered cells endow tissue engineering scaffolds with more biological factors to realize their regenerative functions. Delivering cells with high activities exhibits potential to solve the problem of hypocellularity at the site of defects and is beneficial for the integration between scaffolds and hosts. Even so, its application also might be hindered by lots of challenges. On the one hand, the fate of the implanted exogenous cells was unclear and controversial. The mechanism by which the delivered cells promote tissue regeneration is still needed to be fully studied [92]. Moreover, it is challenging to precisely regulate the behaviors (*i.e.* differentiation, cellular communication) of the implanted cells under the complex *in vivo* environment [93]. Besides, the application of the delivered cells still faces problems of cell sources, preservation, expansion and ethical issues [94, 95]. On the other hand, the 3D printed matrix scaffolds still face many critical issues. For example, the limited resolution of 3D printing approaches has become a bottleneck for constructing the nanostructure for scaffolds. Furthermore, the mechanical properties of many 3D printed scaffolds can hardly match those of host tissues. Therefore, there is still a long way for accurately imitating complex tissues and organ reconstruction. More efforts should be made to propel the development and application of cell-delivery scaffolds by the collaboration between therapist, physiologist, mechanical engineer and biomaterials scientists.
